# Automatic discovery of 100-miRNA signature for cancer classification using ensemble feature selection

**DOI:** 10.1186/s12859-019-3050-8

**Published:** 2019-09-18

**Authors:** Alejandro Lopez-Rincon, Marlet Martinez-Archundia, Gustavo U. Martinez-Ruiz, Alexander Schoenhuth, Alberto Tonda

**Affiliations:** 1 Division of Pharmacology, Utrecht Institute for Pharmaceutical Sciences, Faculty of Science, Utrecht University, David de Wied building,Universiteitsweg 99, Utrecht, 3584 CG The Netherlands; 2 Laboratorio de Modelado Molecular, Bioinformática y diseño de fármacos. Departamento de Posgrado. Escuela Superior de Medicina del Instituto Politécnico Nacional (IPN), Mexico City, Mexico; 3 Faculty of Medicine, National Autonomous University of Mexico; Federico Gomez Children’s Hospital of Mexico, Mexico City, Mexico; 4 Life Sciences and Health, CWI, Amsterdam, Netherlands; 5 UMR 782 GMPA, Université Paris-Saclay, INRA, AgroParisTech, Thiverval-Grignon, France

**Keywords:** MicroRNAs, miRNA, Feature selection, Machine learning, Classifiers, Dataset

## Abstract

**Background:**

MicroRNAs (miRNAs) are noncoding RNA molecules heavily involved in human tumors, in which few of them circulating the human body. Finding a tumor-associated signature of miRNA, that is, the minimum miRNA entities to be measured for discriminating both different types of cancer and normal tissues, is of utmost importance. Feature selection techniques applied in machine learning can help however they often provide naive or biased results.

**Results:**

An ensemble feature selection strategy for miRNA signatures is proposed. miRNAs are chosen based on consensus on feature relevance from high-accuracy classifiers of different typologies. This methodology aims to identify signatures that are considerably more robust and reliable when used in clinically relevant prediction tasks. Using the proposed method, a 100-miRNA signature is identified in a dataset of 8023 samples, extracted from TCGA. When running eight-state-of-the-art classifiers along with the 100-miRNA signature against the original 1046 features, it could be detected that global accuracy differs only by 1.4%. Importantly, this 100-miRNA signature is sufficient to distinguish between tumor and normal tissues. The approach is then compared against other feature selection methods, such as UFS, RFE, EN, LASSO, Genetic Algorithms, and EFS-CLA. The proposed approach provides better accuracy when tested on a 10-fold cross-validation with different classifiers and it is applied to several GEO datasets across different platforms with some classifiers showing more than 90% classification accuracy, which proves its cross-platform applicability.

**Conclusions:**

The 100-miRNA signature is sufficiently stable to provide almost the same classification accuracy as the complete TCGA dataset, and it is further validated on several GEO datasets, across different types of cancer and platforms. Furthermore, a bibliographic analysis confirms that 77 out of the 100 miRNAs in the signature appear in lists of circulating miRNAs used in cancer studies, in stem-loop or mature-sequence form. The remaining 23 miRNAs offer potentially promising avenues for future research.

**Electronic supplementary material:**

The online version of this article (10.1186/s12859-019-3050-8) contains supplementary material, which is available to authorized users.

## Background

Cancer is difficult to diagnose and classify at early stages, and is one of the top leading causes of death worldwide [1]. Therefore, several attempts have been made to identify possible biomarkers for cancer detection. MicroRNAs (miRNAs) represent a class of small noncoding RNA molecules, with a critical role in the post-transcriptional regulation of gene expression. miRNAs also act on several cellular processes, such as cell differentiation, cell cycle progression, and apoptosis. Additionally, in tumors, some miRNAs can function as oncogenes, while others suppress tumors [2]. Succeeding the earliest evidence of miRNA involvement in human cancer by Croce et al. [3], various studies have demonstrated that miRNA expressions are deregulated in human cancer through a variety of mechanisms [4]. Since ectopic modulation of specific miRNAs compromise the hallmarks of cancer, several efforts have been spent to generate scaffold-mediated miRNA-based delivery systems trying to demonstrate the potential of miRNA-mediated therapies.

In comparison to invasive methods currently used for cancer diagnosis, there is an ongoing debate on the use of circulating miRNAs as possible biomarkers due to the fact that they can be detected directly from biological fluids, such as blood, urine, saliva and pleural fluid [5]. MiRNAs possess other qualities of good candidate biomarkers such as: a) they are useful for the identification of cancer types, b) their availability of high-quality measurement techniques for miRNAs and c) they present good conservation between practical and preclinical models [6].

Several studies have shown the properties of miRNAs as oncogenes and tumor suppressors genes [7–9]. Since then, techniques such as microarray (Affymetrix, Agilent) and sequencing techniques (Illumina), have been proposed for their identification [10]. In the context of increasing availability of data, it is of utmost practical importance to build databases of miRNA expressions data for cancer research [11–13] and to extract features that could be used as cancer biomarkers [14–16]. For example, the expression levels of miRNA *hsa-miR-21* change for different cancer types such as: squamous cell lung carcinoma [17], astrocytoma [18], breast cancer [19], and gastric cancer [20]. Following this idea, the scientific community is currently looking for miRNA signatures (a subset of miRNAs), representing the minimal number of miRNAs to be measured for discriminating between different stages and types of cancer.

Thousands of miRNAs have been identified, and currently miRBase (v22.1) contains 1917 stem-loop sequences, and 2657 mature sequences for human microRNA [13]. Although a classification of cancer tumor type is possible using isomirs [21], not all of the miRNAs listed are available in every study, and only a few of them have been shown to work as circulating biomarkers [6]. Obtaining a minimal list of miRNAs able to correctly classify tumors is of utmost practical importance, because it would reduce the measurements needed and improve the likelihood of validation across multiple studies.

Several approaches in the literature propose the use of machine learning techniques for feature selection involving miRNAs. For example, feature selection for identifying miRNA targets [22], for prediction of specific biomarkers for tumor origin [23] and to learn subset of features for tumor classification [24]. In this study, the objective was to use feature selection and to uncover a small miRNAs signature with the aim to correctly classify cancer tumor types, and distinguish between normal and tumor tissue reducing the necessary features by an order of magnitude.

We propose an ensemble feature selection method, starting from a subset of The Cancer Genome Atlas dataset (TCGA) [25], containing 8023 cases, with 28 different types of cancer, and 1046 different stem-loop miRNA expressions (miRBase V16^1^, summarized in Table 10). Typically, classifiers trained on a dataset do not use the whole set of available features to separate classes, but only a subset which could be ordered by relative importance, with a different meaning given to the list by the specific technique, pushing for simpler models. Using 8 state-of-the-art classifiers implemented in the scikit-learn toolbox [26], the most relevant miRNAs are extracted to be used as features for cancer classification. The top *k* features in the list are then evaluated as a potential reduced signature for classification. In this work, after preliminary tests, we select *k*=100 to reduce the original features by an order of magnitude. Because other feature selection methods require the user to specify a desired number of features, this also allows for a fair and meaningful comparison with these methods.

**Fig. 1:**
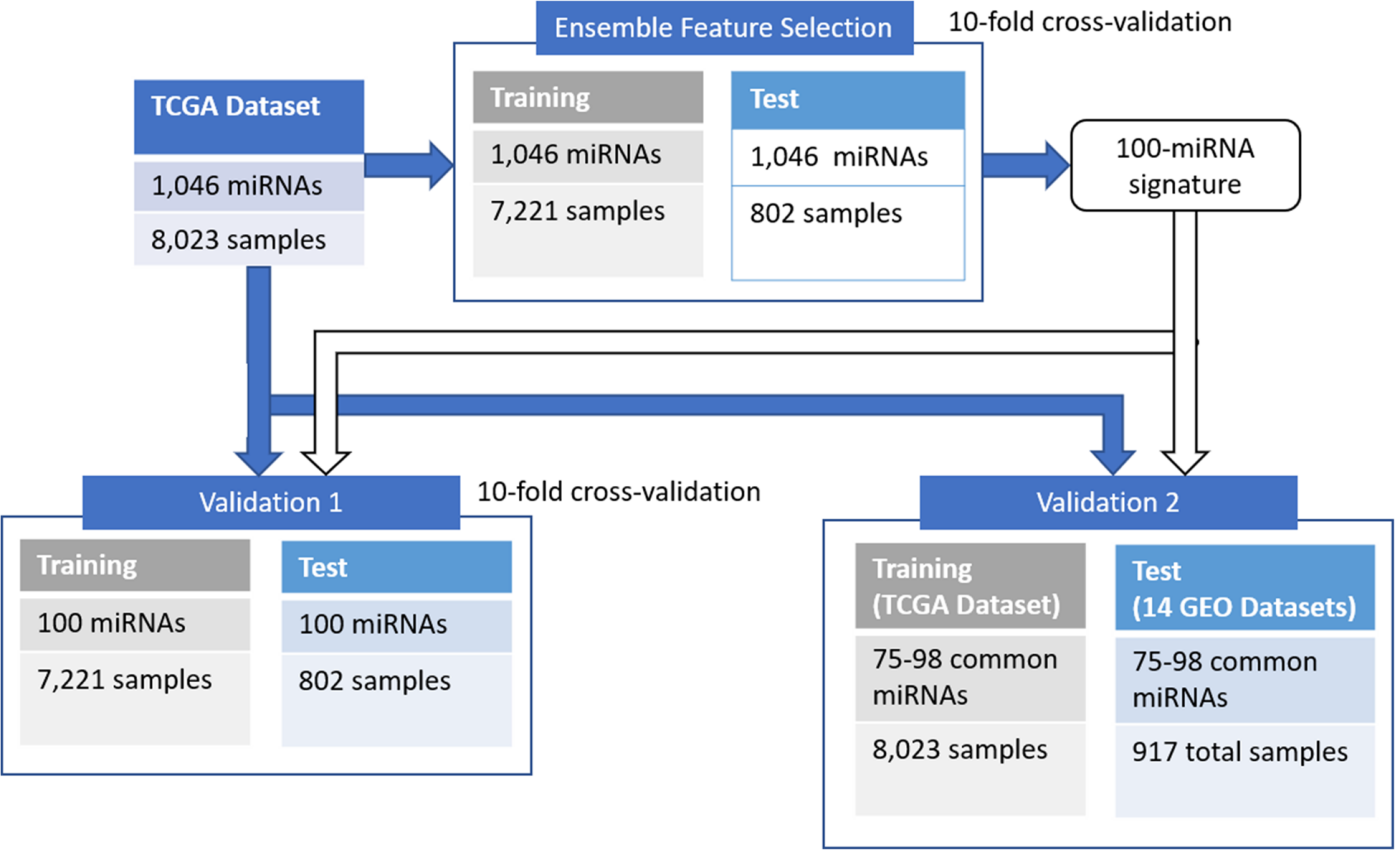
Summary of the different datasets and their use in the experiments

The obtained 100-miRNA signature is first tested to classify the initial TCGA dataset, and later applied on 14 Gene Expression Omnibus (GEO) datasets obtained with different platforms (Affymetrix Multispecies Array miRNA-1, miRNA-2 and miRNA-3, Illumina 2000, and Agilent-021827 Human miRNA Microarray V3), for different cancer tumor types (Prostate, Liver, Breast, Esophageal, Head and Neck Squamous and Lung). A summary of this validation is presented in Fig. 1. Furthermore, the proposed methodology is compared to popular feature selection methods in bioinformatics, such as Univariate Feature Selection, Recursive Feature Elimination, Genetic Algorithms, Least Absolute Shrinkage and Selection Operator, Random Selection, Elastic Net and Ensemble Feature Selection with Complete Linear Aggregation. Next, we use the same signature to try to distinguish molecular subtypes in breast cancer, both for the TCGA dataset and a set of GEO datasets. Finally, the 100 miRNAs included in the signature are evaluated through a meta-analysis based on the medical literature. Because this meta-analysis reveals known relationships between features selected by our approach, relative to the type of cancer considered, it has the potential to yield insight into the biological processes and relationships combinedly affecting miRNAs and cancer.

## Results

### Feature selection and validation on the tCGA dataset

Table 1 compares the classification accuracy on a 10-fold cross-validation for each classifier, using the full 1046 features, and then employing the reduced 100-miRNA signature. It is interesting to notice how the accuracy is, for most cases, unchanged, providing empirical evidence that a 100-miRNA signature is enough to obtain good classification results, with a small statistically significant (T-test, *p*<0.05) difference of 1.4%.

**Table 1:** Accuracy of classifiers used in the experiments on the TCGA dataset

Classifier	Accuracy (10-fold CV)					
	1046 Features		100 Features		Hyper parameters	Feature selection method
	avg	std	avg	std		
Gradient Boosting	0.9398	0.0076	0.9359	0.0086	300 predictors	Decision Trees
Random Forest	0.9351	0.0071	0.9324	0.0073	300 predictors	Decision Trees
Logistic Regression	0.9178	0.0096	0.9237	0.0067	-	Coefficients
Passive Aggressive	0.9117	0.0104	0.8831	0.0115	-	Coefficients
SGD	0.91	0.0074	0.9035	0.0152	-	Coefficients
SVC	0.9211	0.0122	0.9154	0.0065	Linear kernel	Coefficients
Ridge	0.8971	0.0138	0.8305	0.0062	-	Coefficients
Bagging	0.9151	0.0120	0.9110	0.0077	300 predictors	Decision Trees
Average	0.918463	-	0.9044	-	-	-

In the case a classifier is not using standard values for its hyperparameters, the relevant variations are summarized in the corresponding column

**Fig. 2:**
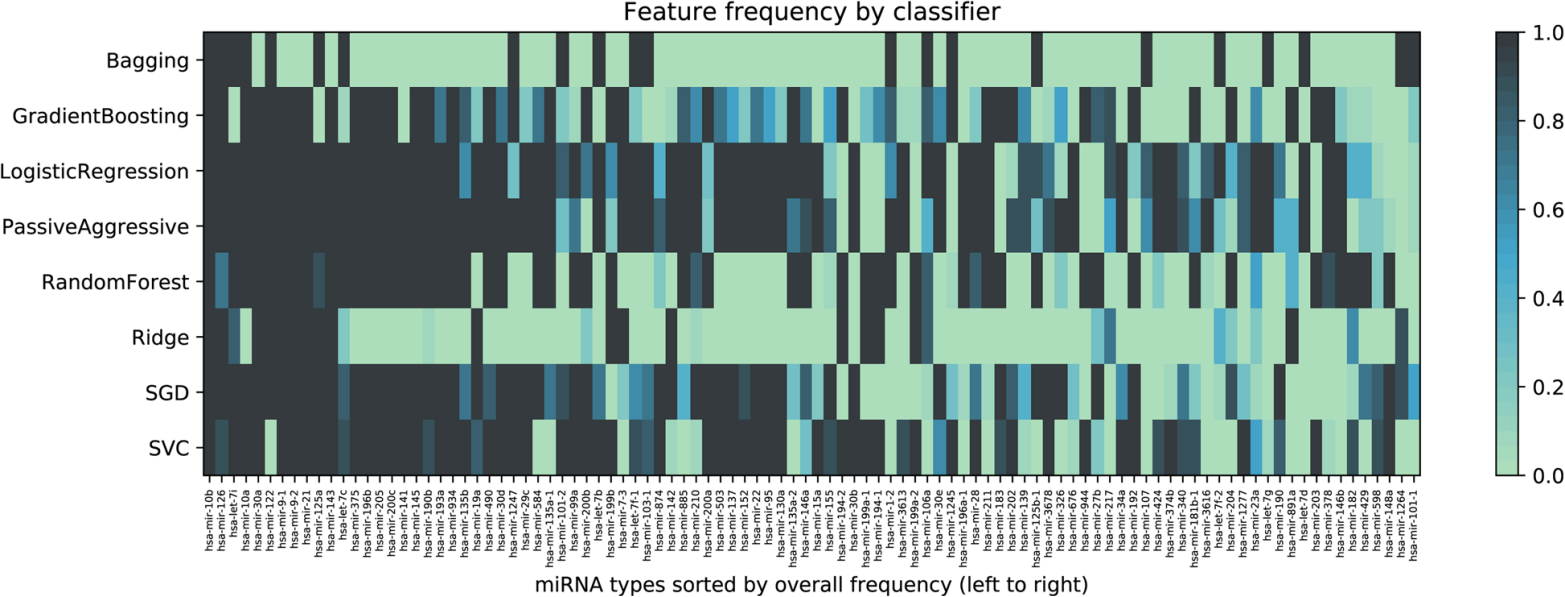
Heatmap with the frequency of the overall top 100 most frequent features, divided by classifier. Features are sorted from overall most to least frequent, from left to right, using information from the whole ensemble. For example, the most frequent is mir-10b, that is considered important by all classifiers. Color intensity is computed using information from instances of the same classifier, only. This shows the different importance that different classifiers assign to each feature

**Fig. 3:**
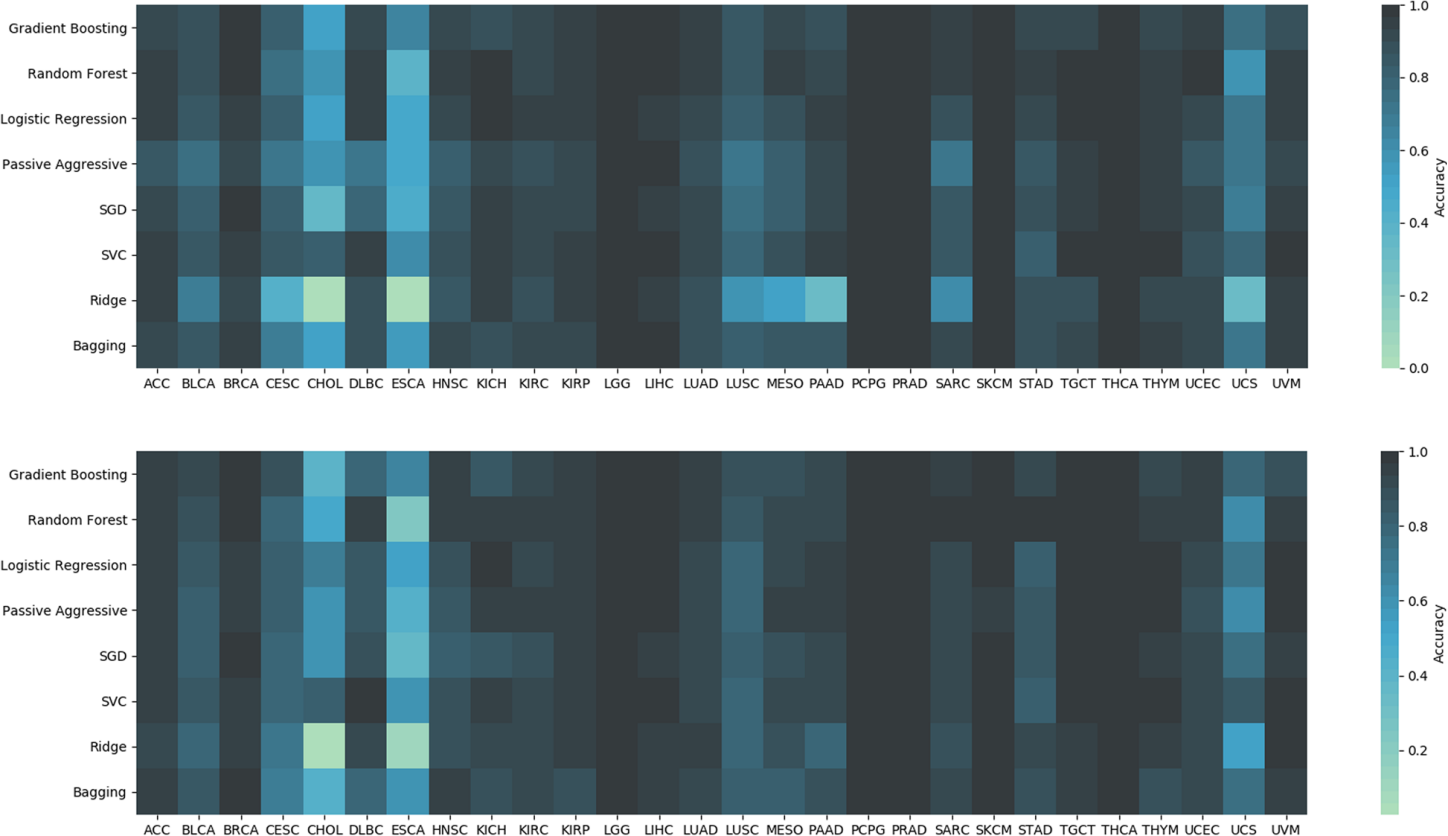
Heatmap of the accuracy by cancer type, by classifier using the 1046 features (top) and the 100-miRNA signature (bottom)

Figure 2 shows a heatmap comparing the relative frequency of the overall top 100 most frequent miRNA features, for each considered classifier. As expected, not all classifiers used the same features to separate the types of cancer, and thus, evaluating their consensus is more robust than just relying upon a single algorithm, as it is commonly accepted in the field of machine learning [27]. It is interesting to notice that while the most common biomarkers appear among the top for most classifier, others make use of only a few. For example, Bagging and Ridge do not use the vast majority of the features exploited by other techniques to discriminate between classes. A further difference between the two classifiers is that features used by Bagging that also appear in the top 100 are clearly important for the classifier, being used in almost 100% of its 10 runs; while it is noticeable how Ridge probably bases its discrimination on features that do not appear among the top 100. This would also explain why Ridge is the only algorithm that presents a decrease in performance when using the 100-miRNA signature. It’s important to note that, while the results emerging from the heatmap suggest that this is indeed the case, Ridge’s decision boundaries should be analyzed more in-depth, for each class and multiple instances, in order to have absolute certainty, a task that is outside of the scope of the current work. Figure 3 shows the difference between 1046 features and 100 features for each cancer type and classifier.

### Normal vs tumor tissue classification

We compared Tumor Tissue (TT) vs Normal Tissue (NT) in a 10-cross fold validation, using stratified cross-validation to maintain the proportions for the two classes inside the folds. The global score and the classification accuracy by class are reported in Table 2. All of the classifiers have fair quality for differentiating between normal tissue and tumor tissue, except Ridge, which is more sensitive to the unbalanced number of examples.

**Table 2:** Accuracy for each classifier in a 10-fold cross-validation for the comparison between Tumor Tissue (TT) and Normal Tissue (NT) for 1046 and 100 features

Classifier	100-NT	100-TT	1046-NT	1046-TT	100-Global	1046-Global
Gradient Boosting	0.8612	0.9944	0.8707	0.9950	0.9846	0.9859
Random Forest	0.8091	0.9978	0.7256	0.9985	0.9839	0.9785
Logistic Regression	0.8423	0.9908	0.8659	0.9764	0.9799	0.9683
Passive Aggressive	0.7177	0.9798	0.8123	0.9728	0.9606	0.9611
SGD	0.8060	0.9902	0.7445	0.9936	0.9767	0.9754
SVC(linear)	0.8517	0.9892	0.8218	0.9771	0.9791	0.9657
Ridge	0.2997	0.9981	0.5994	0.9923	0.9470	0.9635
Bagging	0.8028	0.9953	0.7792	0.9966	0.9812	0.9807

### Comparison to established feature selection methods

Several feature selection techniques have been proposed for microarray data [28]. The most effective approaches include Univariate Feature Selection (UFS), Recursive Feature Elimination (RFE), Elastic Net (EN), Genetic Algorithms (GALGO), Least Absolute Shrinkage and Selection Operator (LASSO) and Ensemble Feature Selection with Complete Linear Aggregation (EFS-CLA). UFS aims at finding the best features, scoring them using univariate statistical tests, such as the ANOVA F-value [29], and ultimately taking the *k* features with the highest scores. RFE runs several times a machine learning algorithm capable of scoring features, such as SVC, iteratively removing the feature with the lowest score [30] until it reaches the user-specified *k* features. EN simply runs the machine learning algorithm Elastic Net [31], and takes the *k* highest-scored features. As Elastic Net is trying to balance accuracy and weight size in a linear model, exploiting L1 and L2 regularization, it is a popular choice for feature selection in bio-informatics [32, 33], because it tends to create sparse models with few weights different from zero. LASSO is a regression analysis method, performing variable selection and regularization to improve prediction accuracy and interpretability of the statistical model it produces [34], so it can be easily used for feature selection, only. All considered feature selection methods are implemented in the machine learning package scikit-learn, already used in the previous experiments. GALGO is a genetic algorithms-based feature selection library in R that ranks the features using several calls to a classifier and choosing the features that appear the most after evolving a subset several times [35]. EFS-CLA is a method that uses instances of SVM with several calls to a subsample of the data, ranks the features by weight value and reduces a percentage at each iteration [36].

As some of these techniques require the user to specify the number of features *k* to be taken, to provide a comparison with the approach presented in this paper, we have selected *k*=100 features using all the formerly described feature selection methods and compared classification accuracy on the considered classifiers with a 10-fold cross validation. For RFE, we have decided to use SVC, as not only it is commonly adopted for feature selection in bioinformatics [30, 37], but also represents a good compromise between accuracy and speed of convergence on our specific dataset. For EN, we have chosen the ElasticNetCV scikit-learn method, which exploits a 3-fold cross-validation to automatically adapt the internal parameter *α*, balancing the importance of L1 and L2 regularization in the model. For the same reasons, the LassoCV scikit-learn method is selected for LASSO. For EFS-CLA, we use percentage of reduction *E*=20*%*, 40 as SVM calls per step, and k =100. Finally, we add a random selection of 100 features, as a baseline reference to portray the efficiency of the feature selection algorithms.

From the results presented in Table 3, it is immediately clear that the 100 features selected by UFS are much less informative than the ones found by the proposed approach. RFE performs better, especially when considering SVC as the classifier used for the cross validation, but overall the performance for the other classifiers is lower. It must also be noted that, among all the methods, RFE is the most computationally expensive, as it calls the considered classifier, SVC in this case, *N*−*k*=1,046−100=946 times, where *N* is the original number of features. All feature selection algorithms, as expected, perform much better than the baseline random selection of features.

**Table 3:** Comparison between different feature selection techniques and the proposed ensemble method for *k*=100, on the TCGA dataset

Classifier	Random	GALGO	EFS-CLA	UFS	EN	LASSO	RFE	EFS
Gradient Boosting	0.8588	0.8782	0.8871	0.9028	0.9208	0.9315	0.9309	0.9359
Random Forest	0.8515	0.8787	0.8824	0.8929	0.9224	0.9341	0.9288	0.9324
Logistic Regression	0.8015	0.8295	0.8832	0.8813	0.8988	0.8996	0.9088	0.9237
Passive Aggressive	0.6986	0.7235	0.8111	0.8091	0.8406	0.8424	0.8506	0.8831
SGD	0.7278	0.764	0.8446	0.8334	0.8649	0.8648	0.8824	0.9035
SVC	0.8077	0.8348	0.8706	0.885	0.9049	0.9008	0.9103	0.9154
Ridge	0.6534	0.6614	0.7422	0.7504	0.7753	0.7751	0.7954	0.8305
Bagging	0.822	0.8382	0.8562	0.8719	0.8889	0.9078	0.9061	0.911
Global Average	0.7777	0.8010	0.8472	0.8534	0.8771	0.8820	0.8892	0.9044
Calls to Classifier	-	60,000	480	-	-	10	946	80

A qualitative analysis of the features selected by each method shows that the highest-scoring ones are easily found by all considered approaches. In particular, from the 100 features found by our approach, 8 are in common with Random, 11 with GALGO, 29 with EFS-CLA, 38 are common to the group obtained through UFS, 44 are shared with the group found by LASSO, 48 again are found by EN, and 54 are in common with RFE.

### Cross-Platform validation on gEO datasets

As different datasets present distinctive sets of miRNAs, it is important to assess the performance of the signature we identified on unseen data. Using the methodology previously described, the proposed approach is validated on the 14 GEO datasets. Each run of a classifier on a dataset was repeated 10 times, to compensate possible random elements that appear during the training phase of specific algorithms, e.g. RandomForest. It is worth noticing how this validation presents considerable challenges. As we are dealing with different platforms, not all of the 100 features in the signature were available everywhere. For most GEO datasets 98 were available, while for GSE62182 featured 75 of them. Furthermore, despite the transformation needed to bring the samples of the GEO datasets in the TCGA dataset space, samples measured by platforms used in the GEO datasets might prove particularly difficult to tackle for classifiers trained on TCGA samples, as most GEO datasets use microarray technology while TCGA uses sequencing. The properties of the used GEO datasets are summarized in Table 4.

**Table 4:** Summary of the used GEO datasets, and the number of features in common with our 100-miRNA signature

Dataset ID	Platform	Tumor Type	#Samples	Total Feats.	Common Feats.	Reference
GSE34496	GPL8786	HNSC	44	847	98	-
GSE36802	GPL8786	PRAD	21	847	98	[38]
GSE67138	GPL8786	LIHC	57	847	98	-
GSE67139	GPL8786	LIHC	115	847	98	-
GSE45604	GPL14613	PRAD	50	2143	98	[39]
GSE48088	GPL14613	BRCA	33	2143	98	[40]
GSE55856	GPL14613	ESCA	108	2143	98	[41]
GSE86277	GPL14613	BRCA	72	2143	98	[42]
GSE116182	GPL14613	LIHC	64	2143	98	-
GSE86278	GPL16384	BRCA	49	3,242	98	[42]
GSE86281	GPL16384	BRCA	50	3,242	98	[42]
GSE31164	GPL10850	LIHC	110	851	98	[43]
GSE105134	GPL10850	BRCA	50	851	98	-
GSE62182	GPL11154	LUAD	94	3,242	75	[44]

**Fig. 4:**
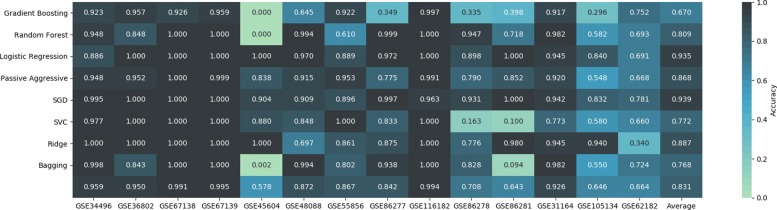
Results with the 100 selected features in the GEO datasets, using a 10-fold cross-validation. From the average accuracy and standard deviation, SGD proves to be significantly better than the rest using a Kolmogorov-Smirnov test (*p*<0.05)

Figure 4 shows the outcomes of the validation for all classifiers. In spite of the difficulties, most algorithms yielded good classification results, with Logistic and SGD in particular featuring over 93% average accuracy on all GEO datasets. Several classifiers, on the other hand, show poor performance on specific datasets, probably due to the way their decision boundaries for that specific class were learned on the TCGA dataset. In this sense, dataset GSE45604 proves to be the overall hardest to classify correctly for most algorithms. GSE86277,GSE86278 and GSE86281, deal with different molecular subtypes of BRCA, that could explain some of the performance issues. Finally the average performance in GSE62182, is because the classifiers have problems differentiating LUAD and LUSC. In general, however, different algorithms seem to have difficulties for different classes and datasets, which suggests that an ensemble approach for classification could compensate local issues.

To the best of our knowledge, the most similar work in literature that we can compare our results to is Telonis et al. [21], where isoform quantification was adopted to classify three of the GEO datasets used in this study (GSE36802, GSE67138, GSE67139), training SVC on a TCGA-derived dataset. For GSE36802, [21] reports an accuracy of 76%, that is surpassed by all of the classifiers. Considering GSE67138, for which an accuracy of 91% is reported, all the algorithms in our case perform better. Finally, for GSE67139, a 96% accuracy, again all the algorithms outperform that value. It must be noted, however, that even this comparison is made difficult by differences in how data was treated: for example, [21] reduced the number of classes to 6 and tested on 4 different types of tumors. In our study, we keep all 28 classes for testing.

### Tumor subtype

To further test our approach, we use the 100-miRNA signature to classify tumor subtypes. As a comparison with GEO datasets is important for our validation, we select molecular subtype in breast cancer (BRCA), as it’s the only tumor class for which molecular subtype information is available in the GEO datasets. From the information in [45, 46], we are able to label 764 of the 777 BRCA samples in the TCGA dataset in 5 different subtypes (Luminal A, Luminal B, Triple-negative/basal-like, HER2-enriched and Normal-like). More information on the subtypes can be found in [47]. Next, we calculate the accuracy in a 10-fold cross validation for the 1046 TCGA features and the 100-miRNA signature, with results reported in Tables 5 and 6 respectively.

**Table 5:** Molecular subtype classification accuracy of Breast Cancer for the 1046 features

	Normal	LumA	LumB	TNBC	Her2	Global
#Samples	33	399	139	135	58	764
Gradient Boosting	0.1818	0.9348	0.5396	0.9333	0.5172	0.7987
Random Forest	0.0606	0.9724	0.4532	0.9630	0.0345	0.7657
Logistic Regression	0.1212	0.8747	0.5540	0.9259	0.4483	0.7606
Passive Aggressive	0.1515	0.8622	0.5612	0.9111	0.4483	0.7539
SGD	0.3030	0.9073	0.4604	0.9556	0.4655	0.7752
SVC	0.2727	0.8797	0.5252	0.9185	0.5345	0.7697
Ridge	0.1515	0.7293	0.4317	0.3704	0.2759	0.5524
Bagging	0.3333	0.9298	0.5108	0.9704	0.4310	0.7973
Average	0.1970	0.8863	0.5045	0.8685	0.3944	0.7467

**Table 6:** Molecular subtype classification accuracy of Breast Cancer for the 100 features

	Normal	LumA	LumB	TNBC	Her2	Global
#Samples	33	399	139	135	58	764
Gradient Boosting	0.2424	0.9248	0.5324	0.9333	0.5517	0.7975
Random Forest	0.2121	0.9599	0.4029	0.9704	0.2069	0.7712
Logistic Regression	0.2727	0.8997	0.4892	0.9037	0.5517	0.7727
Passive Aggressive	0.3939	0.8546	0.4460	0.8667	0.5000	0.7358
SGD	0.4545	0.8897	0.4460	0.8444	0.4310	0.7475
SVC	0.5152	0.8446	0.5108	0.9037	0.5517	0.7581
Ridge	0.0606	0.9474	0.4388	0.8593	0.3966	0.7594
Bagging	0.2727	0.9173	0.4964	0.9481	0.3793	0.7777
Average	0.3030	0.9048	0.4703	0.9037	0.4461	0.7650

The best classification results are obtained for subtypes Triple-Negative Breast Cancer (TNBC) and Luminal A (LumA), due to the scarcity of samples for other subtypes (especially Normal and Her2). Luminal B (LumB) presents considerable similarities to LumA, and the classifiers have difficulty separating the two subtypes using the data at our disposal. For these reasons, and the practical concern that TNBC is the subtype of BRCA with the worst prognosis, we decide to tackle the issue as a binary classification problem, separating TNBC from the other classes. TNBC is a subtype of cancer where the cells have tested negative for estrogen receptors (ER), hormone epidermal growth factor receptor 2 (Her2), and progesterone receptors (PR). This subtype of cancer has limited treatment options and poor prognosis, as hormone therapies or targeted drugs do not work on it. Results of the binary classification problem on TCGA are reported in Table 7.

**Table 7:** TNBC classification from the other molecular subtypes in the TCGA dataset, using 1046 features and 100 signature

	TNBC-100	TNBC-1046	Other-100	Other-1046	Global-100	Global-1046
#Samples	135	135	629	629	764	764
Gradient Boosting	0.9111	0.8963	0.9857	0.9857	0.9725	0.9699
Random Forest	0.8889	0.8815	0.9905	0.9905	0.9725	0.9712
Logistic Regression	0.8963	0.9630	0.9793	0.9587	0.9647	0.9593
Passive Aggressive	0.8815	0.9630	0.9714	0.9523	0.9556	0.9540
SGD	0.8000	0.8222	0.9809	0.9841	0.9490	0.9555
SVC	0.8444	0.8963	0.9666	0.9825	0.9451	0.9673
Ridge	0.8000	0.7259	0.9825	0.9237	0.9503	0.8888
Bagging	0.8444	0.8963	0.9793	0.9825	0.9555	0.9673
Average	0.8583	0.8806	0.9795	0.9700	0.9582	0.9542

Finally, we test the binary subtype classification of BRCA for the GEO datasets, using just the 100-miRNA signature. We create a single dataset composed of 4 series (GSE86281, GSE86277, GSE86278, GSE46823), with 2 classes: TNBC, featuring 139 samples, and all other molecular subtypes (LumA, LumB, and Her2), with 32 samples in total. Using the stem-loop sequences from platform GPL14613, and GPL1368, we use the 98 common stem-loop miRNAs of the 100 in the signature signature for the classification. In Table 8, we show the results of the classification in a 10-fold cross validation, and the accuracy by class.

**Table 8:** Molecular subtype classification of Breast Cancer to separate TNBC from other breast cancer subtypes using the 100-miRNA signature, on the GEO dataset

	TNBC	Other	Global
#Samples	139	44	183
Gradient Boosting	0.9353	0.7500	0.8909
Random Forest	0.9424	0.6136	0.8634
Logistic Regression	0.9065	0.6590	0.8476
Passive Aggressive	0.8561	0.7045	0.8197
SGD	0.9065	0.5227	0.8145
SVC	0.8561	0.7727	0.8355
Ridge	0.8993	0.6136	0.8300
Bagging	0.9496	0.7727	0.9070
Average	0.9065	0.6761	0.8511

### Discussion

The results of the five experiments performed with the 100-miRNA signature (Tumor Type Classification, Tumor Tissue vs Normal Tissue, GEO datasets, BRCA subtype in TCGA, and BRCA subtype in GEO datasets), are reported in Table 9. All classifiers show high levels of accuracy over all trials, with the validation on the GEO datasets (both tumor type and subtype classification) proving to be the hardest task.

**Table 9:** Comparison of the 8 classifiers, for the different experiments with the 100-miRNA signature

		TT vs		TCGA	GEO	
Classifier	TCGA	NT	GEO	(Subtype)	(Subtype)	Global
Gradient Boosting	0.9359	0.9846	0.6697	0.9725	0.8909	0.8907
Random Forest	0.9324	0.9839	0.8085	0.9725	0.8634	0.9121
Logistic Regression	0.9237	0.9799	0.9351	0.9647	0.8476	0.9302
Passive Aggressive	0.8831	0.9606	0.8678	0.9556	0.8197	0.8974
SGD	0.9035	0.9767	0.9393	0.9490	0.8145	0.9166
SVC	0.9154	0.9791	0.7724	0.9451	0.8355	0.8895
Ridge	0.8305	0.9470	0.8867	0.9503	0.8300	0.8889
Bagging	0.9110	0.9812	0.7682	0.9555	0.9070	0.9046

Logistic Regression was the best across all experiments, and Ridge has the worst accuracy

**Table 10:** Summary of the TCGA dataset used in the study

Tumor Type	Acronym	Tumor Tissue	Normal Tissue	Class
Adrenocortical carcinoma	ACC	80	0	0
Bladder Urothelial Carcinoma	BLCA	411	19	1
Breast invasivex carcinoma	BRCA	777	87	2
Cervical squamous cell carcinoma	CESC	306	3	3
Cholangiocarcinoma	CHOL	36	9	4
Lymphoid Neoplasm Diffuse Large B-cell Lymphoma	DLBC	47	0	5
Esophageal carcinoma	ESCA	187	13	6
Head and Neck squamous cell carcinoma	HNSC	487	44	7
Kidney Chromophobe	KICH	66	25	8
Kidney renal clear cell carcinoma	KIRC	260	71	9
Kidney renal papillary cell carcinoma	KIRP	291	34	10
Lower Grade Glioma	LGG	528	0	11
Liver hepatocellular carcinoma	LIHC	374	50	12
Lung adenocarcinoma	LUAD	458	46	13
Lung squamous cell carcinoma	LUSC	341	45	14
Mesothelioma	MESO	86	0	15
Pancreatic adenocarcinoma	PAAD	154	4	16
Pheochromocytoma and Paraganglioma	PCPG	184	3	17
Prostate adenocarcinoma	PRAD	494	52	18
Sarcoma	SARC	260	0	19
Skin Cutaneous Melanoma	SKCM	450	2	20
Stomach adenocarcinoma	STAD	399	45	21
Testicular Germ Cell Tumors	TGCT	156	0	22
Thyroid carcinoma	THCA	513	59	23
Thymoma	THYM	124	2	24
Uterine Corpus Endometrial Carcinoma	UCEC	417	21	25
Uterine Carcinosarcoma	UCS	57	0	26
Uveal Melanoma	UVM	80	0	27
Total		8023	634	

As miRNAs have been shown to regulate approximately 30% of the human genes, and because their dysregulation has been associated with the development and progression of cancer, miRNAs have been found to have the potential to play a critical role in computational oncology. Nevertheless, their analysis and their employment in clinically relevant settings still faces various, specific technical challenges: a) the extremely small size of the miRNAs leads to diverse complications for example with respect to hybridization techniques, b) there is a lack of specificity in detection because of the high similarity of several miRNA family members, and c) the low expression of various miRNAs requires detection methods of utmost sensitivity [48]. To date, most new miRNAs are discovered through cloning, despite these methods being time-consuming, low-throughput, and being biased toward the discovery of abundant miRNAs [49, 50].

Nevertheless, we can conclude from our results that the extracted 100-miRNA signature is able to reliably classify the 28 different types of cancer in the TCGA dataset, and distinguish between normal and tumor tissue. In addition, it is sufficiently stable to be applicable across platforms, such as the ones such as the ones used in the ten GEO datasets and ahich show a good accuracy in differentiating TNBC from other molecular subtypes of BRCA. Looking ahead into the possibility of classifying tumor types using miRNAs, we need to consider circulating miRNAs, and their relationship to cancer studies.

**Fig. 5:**
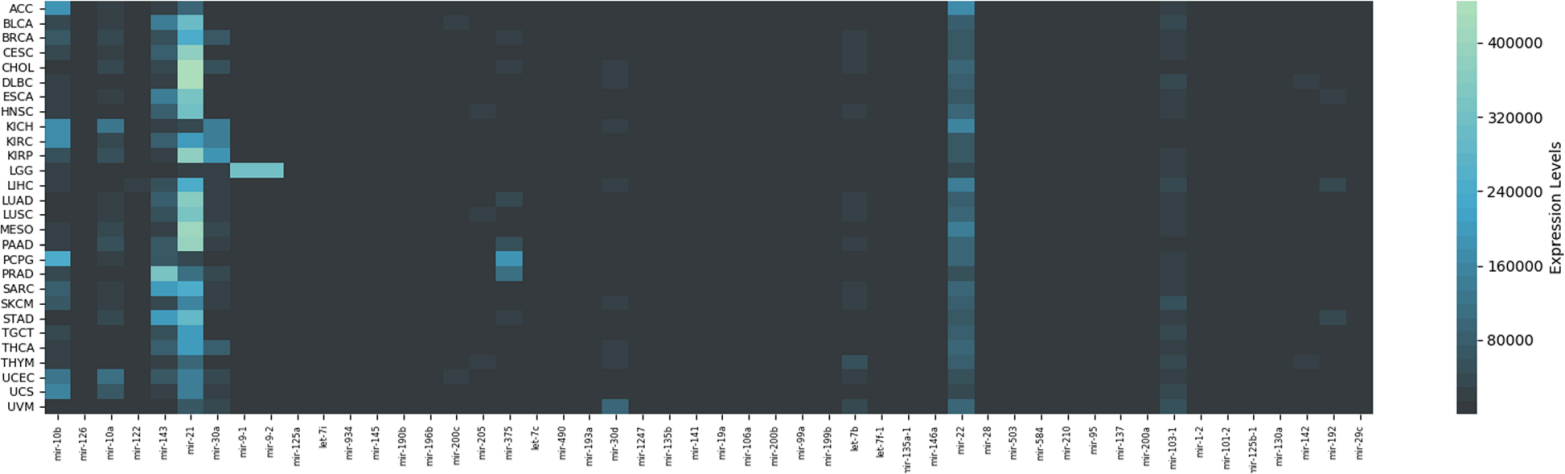
miRNAs mean expression levels (RPMs) of the top 50 miRNAs for each type of cancer tumor tissue

For the miRNAs included in the signature, we performed a bibliographic meta-analysis of specialized literature. The proposed meta-analysis is mainly based on 5 surveys of circulating miRNAs for cancer studies [6, 7, 51–53]. Out of the 100 miRNAs in the signature, 77 appear as circulatory miRNAs, either in their stem-loop form or mature sequence. The complete list for the 100-miRNAs is reported in Annex A of the online Additional file 1, in Fig. 5 shows the expression levels by type of cancer of the top 50 miRNAs.

Across all surveys analyzed, *hsa-miR-21*, included in our signature in stem-loop form, appears to be the most commonly over-expressed miRNA for all classes of tumors, as we would expect of a known oncomarker. In Annex B of the Additional file 1, we present a detailed analysis of the top 50 miRNAs in the signature, showing cancer study type, reference and circulating sample type used for measuring the expression. 23 miRNAs in the signature do not appear in the surveys, but they are mentioned in recent research papers, as promising research leads whose role may need further corroboration (we put the mature sequence as they appear in the study): *miR-211* [54], *miR-135a* [55], *miR-3678-3p* [56], *miR-204* [57], *miR-1228* [58], *miR-374b* [59], *miR-424* [60] *miR-217-5p* [60] *miR-3613-5p* [61], *miR-124* [62], *miR-1277-5p* [63] *miR-190* [64], *miR-934* [65], *miR-490* [66], *miR-1247* [67], *miR-199b* [68], *miR-135a* [55], *miR-503* [69], *miR-584* [70], *miR-137-3p* [71], and *miR-103* [72].

Interestingly, *hsa-mir-135a-1* and *hsa-mir-135a-2*, located inside chromosomes 3 and 12, respectively, generate the same mature active sequence [73]. In the same manner, *hsa-mir-124-1*, *hsa-mir-124-2*, and *hsa-mir-124-3*, generate the same mature sequence *hsa-miR-124-5p*, and *miR-124* is known as a tumor suppressor in head and neck squamous cell carcinoma [74], hepatocellular carcinoma [75] and breast cancer [76]. All of them were identified by our feature selection approach, indicting the presence of miRNA pathways shared across different tumor types. Targeting these miRNA pathways with anti-miRNA-based approaches such as infection with viral particles (having antisense sequence against the specific miRNA) or even drug design of small molecules inhibitors of miRNAs (SMIRs) which can be considered potential anti-tumoral therapy. On the other hand, the down regulation of tumor suppressor miRNAs also contributes to the acquisition of malignant features. For example, by ectopic expression of *hsa-miR-944* which decreases malignant features in gastric [77], colorectal [78] and endometrial [79] cancers. Strikingly, *miR-944* and other understudied miRNAs could have been detected by our approach analizing 28 different types of cancer, suggesting that they could play a key role in the biology of cancer. Future works will include further analyses of the 100-miRNA signature, crossing the information with genetic sources, assessing measures of gene quality and biomarker stability, using tools such as sigQC [80].

## Conclusions

miRNAs fine-tune the regulation of the transcriptome [81, 82]. Alterations in miRNA expression profiles are associated with several diseases, such as cancer. On the other hand, the altered miRNA expression profiles present in cancer could be used as prognostic and/or diagnostic markers. In summary, several miRNA signatures are associated with clinically relevant factors [83, 84]. Therefore, our miRNA signature, which we obtained by using data from different types of cancers, can highlight the presence of so far underestimated miRNA’s such as *miR-944*, and overall has the potential to be used in the frame of microarray based assays, as a potential building block in clinical decision support. Of course, further experimental validation on cancer patient samples will be required to weigh the biological significance of the signature in terms of diagnosing, treating and prognosing the outcome of cancer.

In this study, we developed a new machine-learning approach to obtain a robust, reduced miRNA signature, from a TCGA dataset containing 28 different types of cancer. When tested against other datasets, our system provided good classification accuracy using only the reduced 100-feature signature, despite significant differences in the platforms used to gather the data. A further meta-analysis of literature on the miRNA in the identified signature showed both well-known oncogenic and underestimated miRNA types. The results of this work could potentially be used to uncover new, promising leads of research for a better understanding of miRNA behavior. Furthermore, personal-directed anti-tumoral therapy could be achieved by measurement of the specific, minimal miRNA signature, identified in this work.

## Methods

### Ensemble feature selection

As the objective is to discover and validate a reduced list of miRNAs to be used as a signature for tumor classification, we need to select features that could optimally assist in distinguishing between different cancer types and tumor tissue. In this sense, popular approaches used for feature selection range from univariate statistical considerations, to iterated runs of the same classifier with a progressively reduced number of features in order to assess the contribution of the features to the overall result. As the considered problem is particularly complex, relying upon simple statistical analyses might not suffice. Furthermore, features extracted using an iterative method on one classifier are likely to work well only for that specific classifier. Following the idea behind *ensemble feature selection* [36, 37, 85], we propose the use of multiple algorithms to obtain a more robust and general predictive performance. An ensemble approach has the advantage of obtaining features that will be effective across several classifiers, with a better likelihood of being more representative of the data, and not just of the inner workings of a single classifier.

For this purpose, we train a set of classifiers in order to extract a sorted list of the most relevant features from each. Intuitively, as a feature considered important by the majority of classifiers in the set is also likely to be relevant for our objective, then information from all classifiers is compiled to find the most common relevant features. Starting from a comparison of 22 different state-of-the-art classifiers on the considered dataset, presented in [86], a subset of those classifiers was selected considering both; high accuracy and a way to extract the relative importance of the features from the trained classifier. After preliminary tests to set algorithms’ hyperparameters, 8 classifiers were chosen, all featuring an average accuracy higher than 90% on a 10-fold cross-validation: Bagging [87], Gradient Boosting [88], Logistic Regression [89], Passive Aggressive [90], Random Forest [91], Ridge [92], SGD (Stochastic Gradient Descent on linear models) [93], SVC (Support Vector Machines Classifier with a linear kernel) [94]. All considered classifiers are implemented in the scikit-learn Python toolbox.

Overall, the selected classifiers fall into two broad typologies: those exploiting ensembles of classification trees [95] (Bagging, Gradient Boosting, Random Forest), and those optimizing the coefficients of linear models to separate classes (Logistic Regression, Passive Aggressive, Ridge, SGD, SVC). Depending on classifier typology, there are two different ways of extracting relative feature importance. For classifiers based on classification trees, the features used in the splits are counted and sorted by frequency, from the most to the least common. For classifiers based on linear models, the values of the coefficients associated to each feature can be used as a proxy of their relative importance, sorting coefficients from the largest to the smallest in absolute value. As the two feature extraction methods return heterogeneous numeric values, only the relative sorting of features provided by each classifier was considered. Furthermore, we decide to extract the top 100 most relevant features as a reduction of about an order of magnitude, so we assign to each feature *f* a simple score *s*_*f*_=*N*_*t*_/*N*_*c*_, where *N*_*t*_ is the number of times that specific feature appears among the top 100 of a specific classifier instance, while *N*_*c*_ is the total number of classifiers instances used; for instance, a feature appearing among the 100 most relevant in 73% of the classifiers used would obtain a score *s*_*f*_=0.73. We select 100 features because we wanted to compress the dataset at least 90%, thus from 1046 we reduce it to 100. In order to increase the generality of our results, each selected classifier was run 10 times, using a 10-fold stratified cross-validation, so that each fold preserves the percentage of samples of each class in the original dataset. Thus, *N*_*c*_=80 (8 types of classifiers, run 10 times each). The complete procedure is summarized by Algorithm 1. Different approaches to the aggregation of heterogeneous feature importance from various sources are also possible (see for example [36, 37, 85]), such as assigning to each feature a weight proportional to its relative importance. However, most alternatives would require adding and tuning extra parameters, so we decided to opt for a simpler approach.


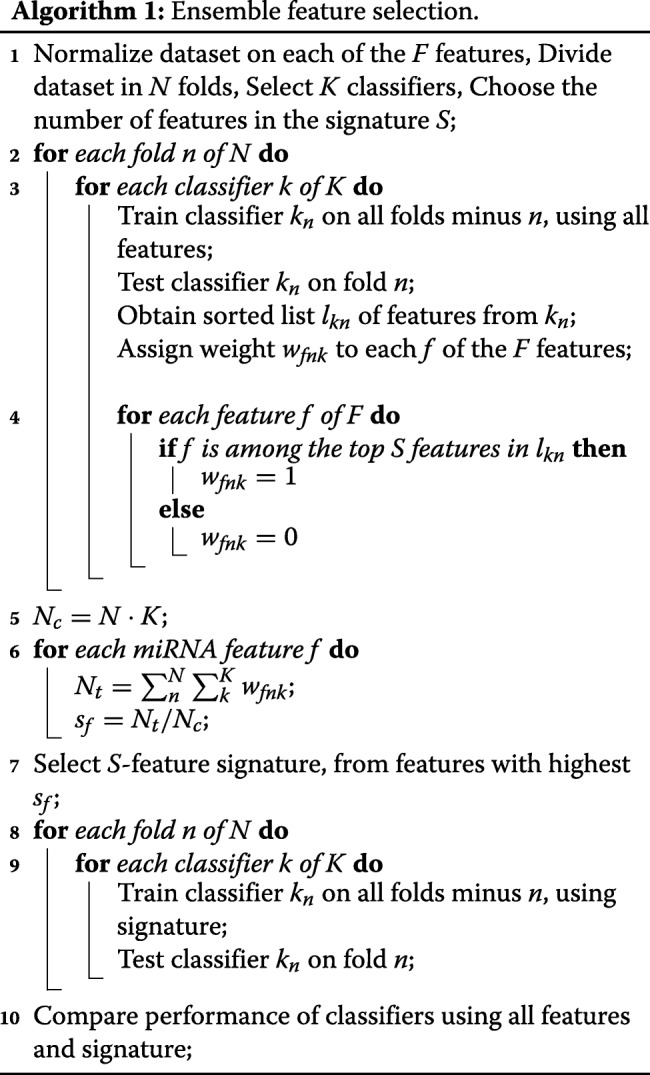


### TCGA dataset

The data was downloaded from the TCGA Data Portal^2^, on September 1, 2016. The used data is miRNA-SEQ files (*.mirna.quantification.txt) a total of 1046 miRNA expression features for each sample in format mirbase V16 for stem-loop sequences^3^. We consider the read per million (RPM) values in the file and we remove all of the samples where the item does not meet the study protocol as stated in the *file annotations*. In summary, the dataset used in the following experiments includes 28 types of tumors, 1046 miRNA features, and 8023 patient samples. Information on the dataset is summarized in Table 10. We standardized the data by removing the mean and scaling to unit variance (specifying that we had learned the standardization on the training set, and applied it to the test set, so that knowledge of the whole dataset did not bias the performance on the test set). In addition, we created a second dataset that differentiates between normal tissue (NT) and tumor tissue (TT) that consists of 8657 samples; 8023 TT and 634 NT.

### Geo datasets

To validate our results, we use 14 datasets from the GEO repository^4^, from 5 different platforms. We use 2 types of miRNA discovery technologies: microarrays and sequencing. miRNAs expression levels are platform and technology dependent [96–98]. Therefore, we need to consider if the information is in stem-loop or mature sequence and then calculate the contributions to make a direct comparison.

In the TCGA dataset, stem-loop sequences were directly measured in raw read counts. When reading a mature sequence, the protocol that was followed assigns a read count to it, and then randomly assigns a read count to one of the stem-loop sequences that share the same mature sequence [99].

#### GPL8786, gPL10850

Affymetrix Multispecies miRNA-1 Array (GPL8786) and Agilent-021827 Human miRNA Microarray V3 (GPL10850) cannot read stem-loop sequences, so the corresponding GEO datasets only show information for mature sequences. Thus, in order to perform a fair comparison, we consider the raw read count for stem-loop sequences as a linear function of the read counts of the mature sequences. If we call the read counts of a specific stem-loop sequence *X*_*i*_, for *hsa-mir-10b* we have for example:

1 $$ {}X_{hsa-mir-10b} = a_{0} \cdot X_{hsa-miR-10b} + a_{1} \cdot X_{hsa-miR-10b*}\\   $$

Where *a*_0_ and *a*_1_ are two coefficients to be set. The mapping between the values of two different platforms *P*1 and *P*2 can then be written as:

2 $$ X_{hsa-mir-10b}^{P1} = a_{2} \cdot X_{hsa-mir-10b}^{P2}   $$

To reduce the problem, we consider only relationships between a stem-loop sequence and its most common corresponding mature sequence e.g hsa-mir-10b to hsa-miR-10b, disregarding hsa-miR-10b*. From Eq. 1 and 2 we then have:

$$ {\begin{aligned} X_{hsa-mir-10b}^{P1} &= a_{2} \cdot X_{hsa-mir-10b}^{P2}\\ X_{hsa-mir-10b}^{P1} &= a_{2} \cdot \left(a_{0} \cdot X_{hsa-miR-10b}^{P2} + a_{1} \cdot X_{hsa-miR-10b*}^{P2}\right)\\ X_{hsa-mir-10b}^{P1} &= a_{2} \cdot a_{0} \cdot X_{hsa-miR-10b}^{P2}\\ X_{hsa-mir-10b}^{P1} &= a^{P}_{hsa-miR-10b} \cdot X_{hsa-miR-10b}^{P2} \end{aligned}}  $$

where $a^{P}_{i}$ becomes the only coefficient to be found, and it represents the transformation between platforms for that specific sequence. A different linear function will be found for each pair of platforms, as we assume that each machine will have unique properties.

For GPL8786 GEO datasets, we consider the linear gene expression values given by the function rmasummary from the Matlab bioinformatics toolbox, which is a normalized robust multi-array average procedure, as a z-score [100, 101]. The equation of a z-score is:

3 $$ Z=\frac{(X-\mu)}{\sigma}  $$

where *X* is the value of a feature; *μ* and *σ* are the average and the standard deviation for a feature. Next, by considering the linear expression values as z-scores, the GEO datasets are mapped to corresponding intensities in the TCGA dataset space, by solving for *X*:

4 $$ X_{i}=\left(Z_{i}\cdot\left(\sigma^{TCGA}_{i}\right)+\mu^{TCGA}_{i}\right)\cdot a^{P}_{i}   $$

where *X*_*i*_ is the intensity of miRNA *i* in the TCGA dataset space, *Z*_*i*_ is the linear gene expression value given by the scaled rmasummary summary function, $\mu ^{TCGA}_{i}$ and $\sigma ^{TCGA}_{i}$ are the average value and the standard deviation for miRNA *i*, both computed on the original TCGA dataset, and $a^{P}_{i}$ is a scale value, dependent on the platform. The value $a^{P}_{i}$ is computed using a subset of all the GEO datasets from the same platform, by minimizing the error between actual class and predicted class, using a model trained in the TCGA dataset with Root Mean Squared Error (RMSE).

5 $$ {\begin{aligned} RMSE= \quad \sqrt{\frac{{\sum\nolimits}_{s=1}^{S}{Predicted_{s}\left(TCGA,a^{P}\right)-Actual_{s}(TCGA)}}{S}} \end{aligned}}  $$

where *S* is the total number of samples in the dataset, and *a*^*P*^ is a vector containing the values of $a^{P}_{i}$ for each feature *i*. A state-of-the-art numerical optimizer [102] is applied to this task, to find the 98 parameters represented by *a*^*P*^.

For GPL10850 we use the MatLab function agferead from the Bioinformatics Toolbox and use the value of gTotalGeneSignal as value for each of the probes and calculate the contributions and $a^{P}_{i}$ as for GPL8786.

#### GPL14613, gPL16384

Affymetrix Multispecies miRNA-2 Array (GPL14613) and Affymetrix Multispecies miRNA-3 Array (GPL16384) measure the stem-loop sequences directly, and denote them by *hp_hsa*. The linear relationship between the TCGA dataset and the corresponding subset of GEO datasets is thus represented by Eq. 2, and the $a^{P}_{i}$ parameters to be found are reduced to the *a*_2*i*_

As remarked by Telonis et al. [21], for these datasets, not all the types of cancer are available, or present the necessary quality standards. Thus, we reduce our analysis to 6 different types of cancer; Prostate, Liver, Breast, Esophageal, Head and Neck Squamous Cell and Lung. For the sequencing data, extra mapping is not necessary besides the sample normalization (platform GPL11154), and we use only stem-loop sequences.

**Fig. 6:**
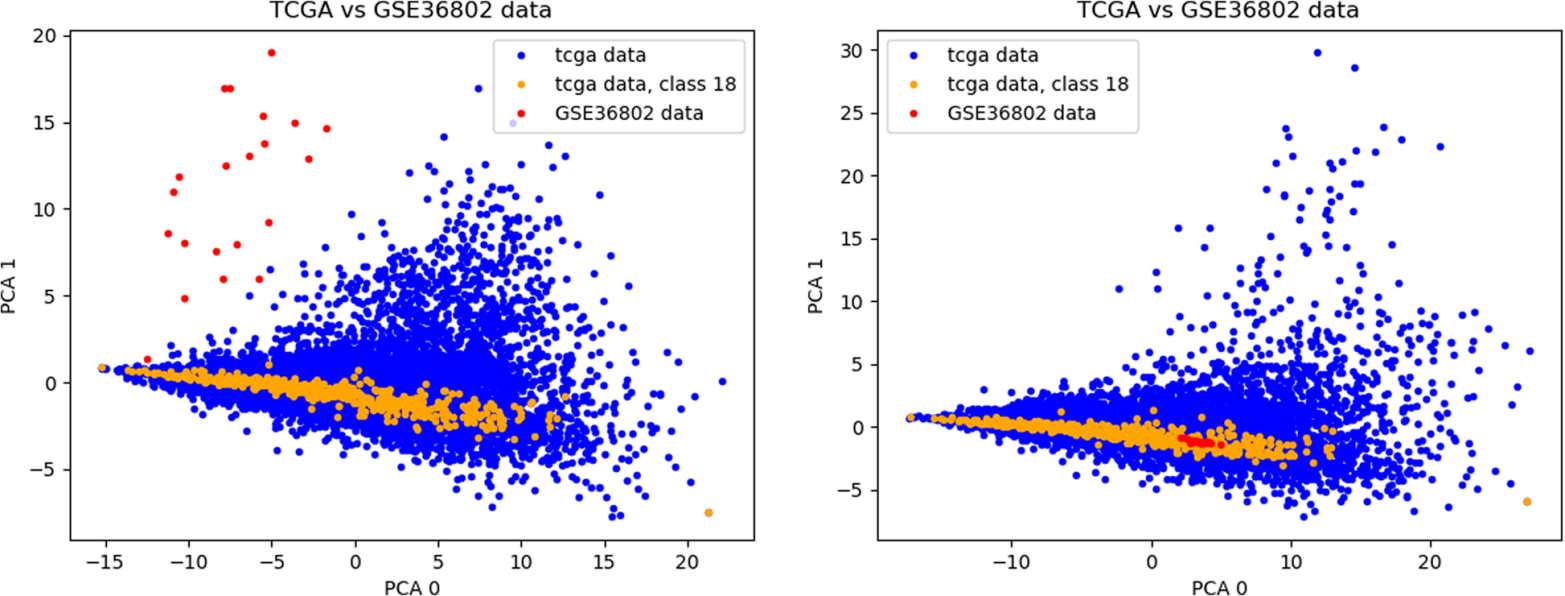
Example of mapping GSE microarray data into TCGA space (GSE36802)

**Fig. 7:**
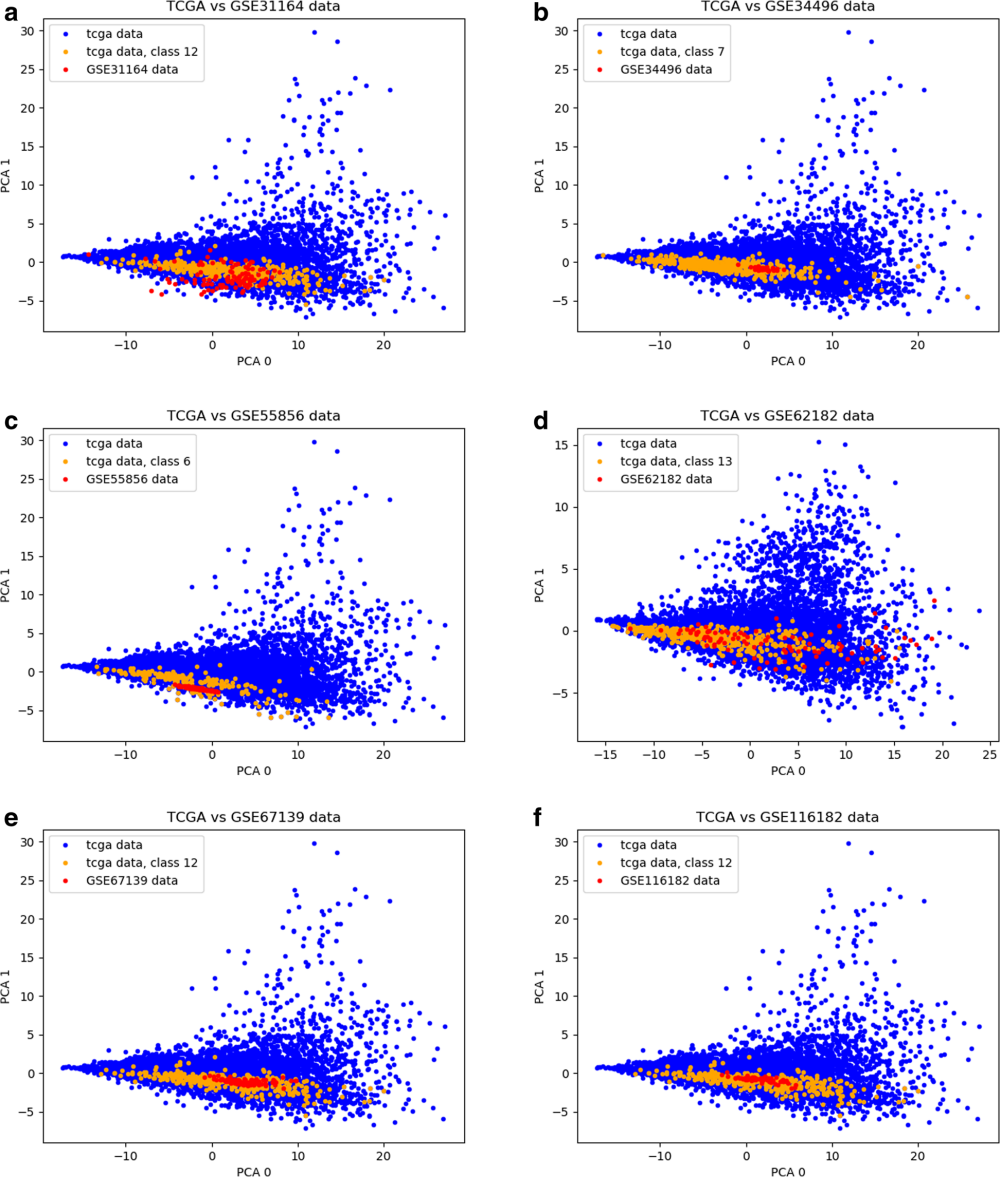
Examples of PCA projections of GEO datasets transformed into the TCGA dataset space. Orange data points represent samples from the target class from the TCGA dataset, the blue data points are other samples in TCGA, and the red points are the projected samples from GEO datasets

Using this procedure, we are able to map the GEO repository measurements into the TCGA dataset space as seen in Fig. 6. Other examples are shown in Fig. 7, where plots were created using the first two dimensions of a Principal Component Analysis (PCA) computed on the TCGA dataset and applied to the GEO datasets, to provide a comparison between the cancer type in each GEO and the corresponding class in TCGA. Remarkably, samples from GEO datasets are often considerably close to samples of the corresponding class in TCGA. During validation, we selected the common features between each GEO dataset and the 100-miRNA signature obtained using the ensemble approach. The accuracy of the classification algorithms was then evaluated by training them on the TCGA dataset and testing them on each GEO dataset. A summary of the experiments is presented in Fig. 1.

## Additional file

Additional file 1 Annex B-Table comparing the top 50 most frequent features extracted by the machine learning algorithms with existing biomarkers references in literature.
Annex C- Figure with all PCA projections of GEO datasets.
Annex D- Explanation of n-fold cross-validation. (PDF 505 kb)

## Data Availability

The code and the datasets are available at https://github.com/steppenwolf0/miRNAs100

## Abbreviations

ACC: Adrenocortical carcinoma
BLCA: Bladder Urothelial carcinoma
BRCA: Breast invasive carcinoma
CESC: Cervical squamous cell carcinoma
CHOL: Cholangiocarcinoma
DLBC: Lymphoid neoplasm diffuse large B-cell lymphoma
EFS-CLA: Ensemble feature selection with complete linear aggregation
EN: Elastic net
ESCA: Esophageal carcinoma
GEO: Gene expression omnibus
HNSC: Head and neck squamous cell carcinoma
KICH: Kidney chromophobe
KIRC: Kidney renal clear cell carcinoma
KIRP: Kidney renal papillary cell carcinoma
LASSO: Least absolute shrinkage and selection operator
LGG: Lower grade glioma
LIHC: Liver hepatocellular carcinoma
LumA: Luminal A
LUAD: Lung adenocarcinoma
LumB: Luminal B
LUSC: Lung squamous cell carcinoma
MESO: Mesothelioma
miRNA: microRNA
NT: Normal tissue
PAAD: Pancreatic adenocarcinoma
PCA: Principal component analysis
PCPG: Pheochromocytoma and paraganglioma
RMSE: Root mean squared error
PRAD: Prostate adenocarcinoma
RFE: Recursive feature elimination
RPM: Read per million
SARC: Sarcoma
SGD: Stochastic gradient descent
SKCM: Skin cutaneous melanoma
STAD: Stomach adenocarcinoma
SVC: Support vector machines classifier
TCGA: The cancer genome atlas
TGCT: Testicular germ cell tumors
THCA: Thyroid carcinoma
THYM: Thymoma
TNBC: Triple negative breast cancer
TT: Tumor tissue
UCEC: Uterine corpus endometrial carcinoma
UCS: Uterine carcinosarcoma
UFS: Univariate feature selection
UVM: Uveal melanoma

## Footnotes

1 ftp://mirbase.org/pub/mirbase/16/

2 https://tcga-data.nci.nih.gov/docs/publications/tcga/

3 ftp://mirbase.org/pub/mirbase/16/genomes/hsa.gff

4 https://www.ncbi.nlm.nih.gov/gds
