# Supplementary material for: Automatic discovery of 100-miRNA signature for cancer classification using ensemble feature selection
Source: BMC Bioinformatics. 2019 Sep 18;20:480. doi: 10.1186/s12859-019-3050-8 (PMC6751684; doi:10.1186/s12859-019-3050-8)
Supplement: Supplementary file 1 — Annex B-Table comparing the top 50 most frequent features extracted by the machine learning algorithms with existing biomarkers references in literature. Annex C- Figure with all PCA projections of GEO datasets. Annex D- Explanation of n-fold cross-validation. (PDF 505 kb) [file 12859_2019_3050_MOESM1_ESM.pdf]

Supplementary Data

Annex A

List of the top 100 most relevant features identified by the proposed methodology, in order of importance:

hsa-mir-10b	hsa-mir-19a	hsa-mir-30e	hsa-mir-1228
hsa-mir-126	hsa-mir-106a	hsa-mir-7-3	hsa-mir-146b
hsa-mir-10a	hsa-mir-200b	hsa-mir-885	hsa-mir-15a
hsa-mir-122	hsa-mir-99a	hsa-mir-152	hsa-let-7g
hsa-mir-143	hsa-mir-199b	hsa-mir-211	hsa-mir-148a
hsa-mir-21	hsa-let-7b	hsa-mir-135a-2	hsa-mir-182
hsa-mir-30a	hsa-let-7f-1	hsa-mir-194-2	hsa-mir-196a-1
hsa-mir-9-1	hsa-mir-135a-1	hsa-mir-30b	hsa-mir-199a-2
hsa-mir-9-2	hsa-mir-146a	hsa-mir-378	hsa-mir-374b
hsa-mir-125a	hsa-mir-22	hsa-mir-181b-1	hsa-mir-424
hsa-let-7i	hsa-mir-28	hsa-mir-183	hsa-let-7d
hsa-mir-934	hsa-mir-503	hsa-mir-23a	hsa-mir-944
hsa-mir-145	hsa-mir-584	hsa-mir-202	hsa-mir-203
hsa-mir-190b	hsa-mir-210	hsa-mir-3678	hsa-mir-217
hsa-mir-196b	hsa-mir-95	hsa-mir-194-1	hsa-mir-328
hsa-mir-200c	hsa-mir-137	hsa-mir-204	hsa-mir-3613
hsa-mir-205	hsa-mir-200a	hsa-mir-34a	hsa-mir-124-2
hsa-mir-375	hsa-mir-103-1	hsa-mir-155	hsa-mir-124-3
hsa-let-7c	hsa-mir-1-2	hsa-mir-708	hsa-mir-1277
hsa-mir-490	hsa-mir-101-2	hsa-mir-1245	hsa-mir-139
hsa-mir-193a	hsa-mir-125b-1	hsa-mir-874	hsa-mir-190
hsa-mir-30d	hsa-mir-130a	hsa-mir-199a-1	hsa-mir-29b-1
hsa-mir-1247	hsa-mir-142	hsa-mir-221	hsa-mir-30c-2
hsa-mir-135b	hsa-mir-192	hsa-mir-27b	hsa-let-7f-2
hsa-mir-141	hsa-mir-29c	hsa-mir-340	hsa-mir-124-1

Annex B

Table 1. Table comparing the top 50 most frequent features extracted by the machine learning algorithms with existing biomarkers references in literature either in stem-loop or mature sequence. **BRCA** Breast Cancer, **CRC** Colorectal Cancer,**LC** Lung Cancer, **PAAD** Pancreatic Cancer, **OV** Ovarian Cancer, **ESCA** esophageal squamous cell carcinoma, **HC** Hematological Cancer ,**DLBC** Diffuse large B-cell lymphoma, **OSCC** oral squamous cell carcinoma, **MM** Multiple myeloma ,**NPC** Nasopharyngeal cancer, **HNSC** neck squamous cell carcinoma, **OS** Osteosarcoma, **CHOL** Cholangiocarcinoma, **UT** Urinary tract ,**GBM** Glioblastoma, **CNSL** Central nervous system lymphoma, **MA** Melanoma, **PE** Pleural effusion ,**PB** Pereipheral Blood, **PJ** Pacreatic Juice.

miRNAs	Plasma	Serum	Urine	Blood	Sputum	Saliva	Stool	PE	PJ
miR-10b	BRCA [1] [2], CRC [1], PAAD [1],	BRCA [1], LC [1],							
miR-126	BRCA [1], LC [1] [2],	BRCA [1],	BLCA [3],	LC [3], OV [3],	LC [3],				
miR-10a	LC [1],	HC [2],		BRCA [1], ESCA [3],					
miR-122	GC [1] [2], HCC [1],	HCC [1], Liver [2],		CRC [1],					
miR-143	CRC [1], PAAD [1],	HCC [1], BRCA [1], PCA [1],		GC [3],					
miR-21	CHOL [4], BRCA [1] [4], CRC [1], HL [4], Liver [2], LC [1] [4] [2], ESCA [4], OS [4], PAAD [1] [4], HC [2], Neck [4], PCA [1] [4], HCC [1], MA [4], GC [1] [4] [2], GBM [4], NPCA [4],	BRCA [1] [4] [2], CNSL [4], HC [2], HCC [1], Liver [4] [2], PCA [1] [4] [2], GBM [4], PAAD [1] [4], PCA [4], LC [1] [4] [2], OV [4], DLBC [4], GC [1] [4], CRC [1] [4] [2],	BRCA [4],	BRCA [3], DLBC [3], LC [3], OV [3], GC [3] [2], GC [1](PB), LC [1](PB),	LC [3], LC [4](BAL+),	ESCA [4], PAAD [4],	PAAD [4],		
miR-30a	BRCA [1] [2], LC [4],	ESCA [4](Exo),		BRCA [1],					
	LC [1],								
miR-9-1		PCA [5]							
miR-9-2		PCA [5]							
miR-125a		LC [4], BRCA [4], Liver [4],				OSCC [3], Oral [4],			
let-7i	PAAD [1], OV [4],	BRCA [1], GC [4], LC [4], OV [4],							
miR-934				BRCA [6],PCA [6]					
miR-145	BRCA [1] [2], CRC [1], LC [1], PCA [1],	BRCA [1] [2], CRC [2], LC [1],		BRCA [1],	LC [3],				
miR-190b	LC [1],			LC [1],					
miR-196b		PAAD [1],							
miR-200c	BRCA [1], CRC [1] [4],	CRC [1] [4] [2], ESCA [4], GC [4], LC [4], OV [4], PAAD [1], PCA [1] [2], OV [4](Exo),		BRCA [4], BRCA [1], OV [3], GC [1] [4] [2],					
miR-205	LC [2],	BRCA [1], LC [1] [2],		OV [3],	LC [3],				PAAD [1],
miR-375	CRC [4], LC [1] [4], ESCA [4], PAAD [1], PCA [1] [4],	BRCA [4], GC [1] [2], Liver [4] [2], LC [4], ESCA [4],	PCA [2],	PCA [3],	LC [3],				
let-7c	LC [4], PCA [1],	BRCA [4], GC [4],		BRCA [3], MA [3],					
miR-490	MM [7]								
miR-193a				BRCA [1], CRC [1] [2],					
miR-30d		LC [1] [4] [2],		LC [3],				GC [3], LC [3],	
miR-1247									PAAD [8]
miR-135b	CRC [5]								
miR-141	CRC [4] [2], PAAD [1], PCA [1], PCA [4](Ves),	LC [1] [4], OV [4], PCA [1] [4] [2], UT [4], OV [4](Exo), PCA [4](Exo),	PCA [2],	BRCA [4], OV [3], PCA [3],					
miR-19a	BLCA [4], CRC [1], LC [1] [2], MA [4],	BRCA [4], CRC [4], LC [4], MM [4],							
miR-106a	CRC [4], GC [1] [4] [2], PAAD [1],	CRC [4], LC [1] [2],		GC [3], GC [1](PB),			CRC [4],		
miR-200b	PCA [4],	OV [4], PAAD [1], PCA [4], OV [4](Exo),		OV [3], PAAD [3],	LC [3],				
	LC [1],								
miR-99a		PAAD [1],							
miR-199b		UT [9]							
let-7b	BRCA [2], LC [4],	BRCA [1], HCC [4], LC [4], OV [4],							
let-7f-1	LC [1] [2], OV [4], LC [4](Ves),	GC [4], HCC [4], Liver [2],		LC [1],			CRC [4],		
miR-135a-1							CRC [10]		
miR-146a		PAAD [1],							
miR-22	PAAD [1],	PAAD [1],		LC [1], ESCA [3],				LC [1],	
miR-28		LC [5]							
miR-503	GC [11]								
miR-584	LC [12]								
miR-210	BRCA [1] [4], LC [1] [2], PAAD [1] [4],	BLCA [4], BRCA [1], DLBC [4], HC [2], Liver [4], PAAD [1], PCA [1] [2], Renal [4], Glioma [4],	BLCA [4],	DLBC [3], PAAD [3],	LC [3],	PAAD [4],			PAAD [4], PAAD [1],
miR-95	CRC [5]								
miR-137	HNSC [13]								
miR-200a		PCA [1],PAAD [1]							
miR-103-1		BLCA [14]							
miR-1-2		BRCA [1],HCC [1]							
miR-101-2		BRCA [1] [2], HCC [1], Liver [2],							
miR-125b-1		LC [1],BRCA [1],PCA [1]							
miR-130a				CRC [1]					
miR-142	CRC [1]								
miR-192	GC [1] [2], CRC [1], HCC [1],								
miR-29c		CRC [1] [4] [2], LC [1] [4] [2], NPCA [4], OS [4],							

Annex C

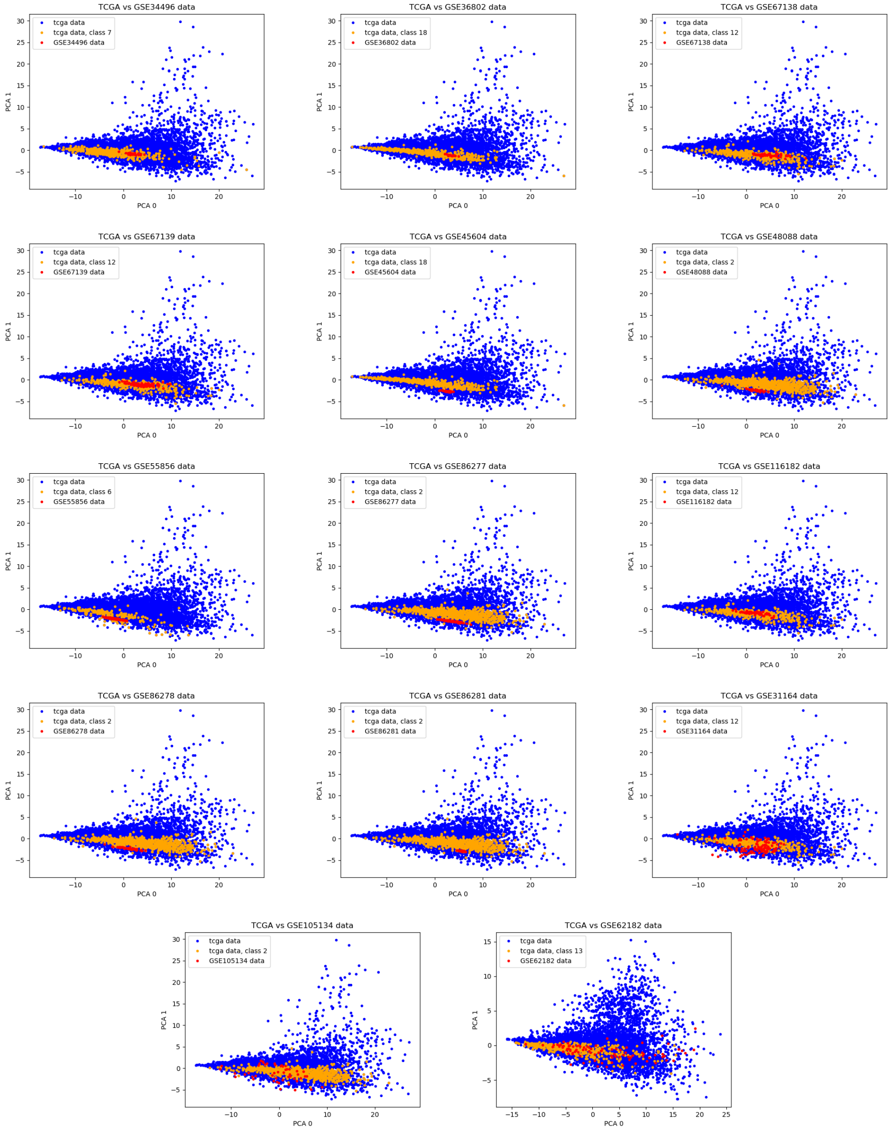

Fig 1. PCA projections of GEO datasets transformed into the TCGA dataset space. Orange data points represent samples from the target class from the TCGA dataset, the blue data points are other samples in TCGA, and the red points are the projected samples from GEO datasets.

Annex D

Classifiers are supervised machine learning algorithms that are able to learn how to separate samples in classes, based on the values of their features. Classifiers implicitly learn the properties of classes starting from training data, that is, a dataset containing samples already associated to their respective class. Once trained, classifiers are in principle able to generalize, and associate unseen samples to known classes.

A popular measurement used to assess the quality of a classifier is termed *accuracy*. Basically, given a dataset for which the correct assignment of samples to classes is known, accuracy measures the percentage of times that a classifier is correct in predicting the class of a sample, comparing the prediction to its known class.

In order to properly assess the generalization ability of a classifier, it is essential to test it properly, as most machine learning algorithms tend to overfit the training data, that is, learning relationships that only exist in the training data, and thus generalize poorly. The basic methodology for testing a classifier is to separate all available labeled data into a training and a test set, train the classifier on the training set, and then test it on the test set, evaluating the final accuracy.

A more refined approach is a *k-fold cross-validation*: the available data is randomly split into k parts, called *folds*. The following procedure is repeated for k iterations: at iteration i, the classifier is trained on all available folds except the i-th, and then tested on the i-th. The accuracy for each fold is stored, and ultimately averaged over all folds, thus providing the user with a more rigorous assessment of the classifier's appropriateness to the current problem.

References

1. He Y, Lin J, Kong D, Huang M, Xu C, Kim TK, et al. Current state of circulating microRNAs as cancer biomarkers. Clinical chemistry. 2015; p. clinchem–2015.
2. Cheng G. Circulating miRNAs: roles in cancer diagnosis, prognosis and therapy. Advanced drug delivery reviews. 2015;81:75–93.
3. Calore F, Lovat F, Garofalo M. Non-coding RNAs and cancer. International journal of molecular sciences. 2013;14(8):17085–17110.
4. Larrea E, Sole C, Manterola L, Goicoechea I, Armesto M, Arestin M, et al. New concepts in cancer biomarkers: circulating miRNAs in liquid biopsies. International journal of molecular sciences. 2016;17(5):627.
5. Wang J, Zhang KY, Liu SM, Sen S. Tumor-associated circulating microRNAs as biomarkers of cancer. Molecules. 2014;19(2):1912–1938.
6. Zhao H, Shen J, Hu Q, Davis W, Medico L, Wang D, et al.. Effects of preanalytic variables on circulating microRNAs in whole blood; 2014.
7. Jiang Y, Luan Y, Chang H, Chen G. The diagnostic and prognostic value of plasma microRNA-125b-5p in patients with multiple myeloma. Oncology letters. 2018;16(3):4001–4007.
8. Wang J, Raimondo M, Guha S, Chen J, Diao L, Dong X, et al. Circulating microRNAs in pancreatic juice as candidate biomarkers of pancreatic cancer. Journal of Cancer. 2014;5(8):696.

9. Montalbo R, Izquierdo L, Ingelmo-Torres M, Lozano JJ, Capitán D, Alcaraz A, et al. Prognostic value of circulating microRNAs in upper tract urinary carcinoma. Oncotarget. 2018;9(24):16691.

10. Koga Y, Yasunaga M, Takahashi A, Kuroda J, Moriya Y, Akasu T, et al. MicroRNA expression profiling of exfoliated colonocytes isolated from feces for colorectal cancer screening. Cancer prevention research. 2010; p. 1940–6207.

11. Shin VY, Ng EK, Chan VW, Kwong A, Chu KM. A three-miRNA signature as promising non-invasive diagnostic marker for gastric cancer. Molecular cancer. 2015;14(1):202.

12. Wang H, Peng R, Wang J, Qin Z, Xue L. Circulating microRNAs as potential cancer biomarkers: the advantage and disadvantage. Clinical epigenetics. 2018;10(1):59.

13. Hsu CM, Lin PM, Wang YM, Chen ZJ, Lin SF, Yang MY. Circulating miRNA is a novel marker for head and neck squamous cell carcinoma. Tumor Biology. 2012;33(6):1933–1942.

14. Jiang X, Du L, Duan W, Wang R, Yan K, Wang L, et al. Serum microRNA expression signatures as novel noninvasive biomarkers for prediction and prognosis of muscle-invasive bladder cancer. Oncotarget. 2016;7(24):36733.
